# Dysfunction of Foxp3^+^ Regulatory T Cells Induces Dysbiosis of Gut Microbiota via Aberrant Binding of Immunoglobulins to Microbes in the Intestinal Lumen

**DOI:** 10.3390/ijms24108549

**Published:** 2023-05-10

**Authors:** Kouhei Koshida, Mitsuki Ito, Kyosuke Yakabe, Yoshimitsu Takahashi, Yuki Tai, Ryouhei Akasako, Tatsuki Kimizuka, Shunsuke Takano, Natsumi Sakamoto, Kei Haniuda, Shuhei Ogawa, Shunsuke Kimura, Yun-Gi Kim, Koji Hase, Yohsuke Harada

**Affiliations:** 1 Laboratory of Pharmaceutical Immunology, Faculty of Pharmaceutical Sciences, Tokyo University of Science, 2641 Yamazaki, Noda 278-8510, Japan; k.koshida516@keio.jp (K.K.); 3a23701@ed.tus.ac.jp (M.I.);; 2 Division of Biochemistry, Faculty of Pharmacy and Graduate School of Pharmaceutical Sciences, Keio University, Tokyo 105-8512, Japankimura-sn@pha.keio.ac.jp (S.K.); hase-kj@pha.keio.ac.jp (K.H.); 3 Research Center for Drug Discovery, Faculty of Pharmacy and Graduate School of Pharmaceutical Sciences, Keio University, Tokyo 105-8512, Japan; ykim@keio.jp; 4 Department of Immunology, University of Toronto, Toronto, ON M5S 1A8, Canada; 5 Division of Integrated Research, Research Institute for Biomedical Sciences, Tokyo University of Science, Noda 278-0022, Japan; shugyaba@rs.tus.ac.jp; 6 The Institute of Fermentation Sciences (IFeS), Faculty of Food and Agricultural Sciences, Fukushima University, Fukushima 960-1296, Japan

**Keywords:** Foxp3^+^ regulatory T cell, gut microbiota, immunoglobulin G, immunoglobulin A, helper T cell

## Abstract

Foxp3^+^ regulatory T (Treg) cells prevent excessive immune responses against dietary antigens and commensal bacteria in the intestine. Moreover, Treg cells contribute to the establishment of a symbiotic relationship between the host and gut microbes, partly through immunoglobulin A. However, the mechanism by which Treg cell dysfunction disturbs the balanced intestinal microbiota remains unclear. In this study, we used Foxp3 conditional knockout mice to conditionally ablate the *Foxp3* gene in adult mice and examine the relationship between Treg cells and intestinal bacterial communities. Deletion of *Foxp3* reduced the relative abundance of Clostridia, suggesting that Treg cells have a role in maintaining Treg-inducing microbes. Additionally, the knockout increased the levels of fecal immunoglobulins and immunoglobulin-coated bacteria. This increase was due to immunoglobulin leakage into the gut lumen as a result of loss of mucosal integrity, which is dependent on the gut microbiota. Our findings suggest that Treg cell dysfunction leads to gut dysbiosis via aberrant antibody binding to the intestinal microbes.

## 1. Introduction

Forkhead box p3 (Foxp3) is the master transcription factor of regulatory T (Treg) cells, which are crucial for immune tolerance [1,2,3,4]. Scurfy mice have a missense mutation in the *Foxp3* gene and therefore develop severe systemic autoimmune inflammatory diseases [5]. In humans, mutations in the *FOXP3* gene lead to immune dysregulation, polyendocrinopathy, enteropathy, and X-linked syndrome, which, in turn, results in fatal lymphoproliferation and widespread multiorgan autoimmunity, including autoimmune enteropathy, dermatitis, thyroiditis, and type I diabetes [6,7,8]. There are two types of Foxp3^+^ Treg cells: thymus-derived Treg (tTreg) cells, which develop in the thymus, and peripherally derived Treg (pTreg) cells, which develop from naïve CD4^+^ T cells in the periphery. pTreg cells develop in response to antigens derived from gut microbiota and food and are predominant in the intestinal tissues. Many studies have indicated that pTreg cells are critical for immune tolerance against these antigens [9,10,11,12].

The mammalian intestinal tract harbors trillions of commensal bacteria that constitute a complex ecosystem called the gut microbiota. The gut microbiota is essential for hosts to obtain essential metabolites, inhibit colonization of pathogens, and reinforce intestinal barrier functions. The symbiotic relationship between the host and gut commensal bacteria is partly established by antibodies, particularly immunoglobulin (Ig)A, which is produced by B cells in response to intestinal bacteria [13,14]. IgA is transported through the epithelium into the intestinal lumen by the polymeric immunoglobulin receptor (pIgR) and plays a fundamental role in maintaining a balanced and diverse gut microbiota and promoting the elimination of pathogenic bacteria. Although IgG is the most abundant antibody subclass in the bloodstream, a relatively small amount of IgG is found in the intestinal lumen, which is transported across the intestinal epithelium by the neonatal Fc receptor (FcRn) and contributes to pathogen clearance [15]. In addition, pathogen-specific maternal IgG in breast milk confers protection to neonates against intestinal infection [16,17]. Although the protective role of mucosal IgG against pathogenic bacterial infection has been investigated [18,19], a role in promoting a symbiotic relationship between the host and the gut microbiota has not yet been established.

Alterations in the composition of the gut microbiota are associated with various diseases, such as inflammatory bowel disease (IBD), diabetes, obesity, and allergies [20,21,22]. Treg cell deficiency has been shown to cause dysbiosis of the gut microbiota. It has been reported that Treg cell-deficient scurfy mice develop gut dysbiosis and that antibiotic treatment suppresses the associated lethal inflammation [23,24,25]. This gut dysbiosis in these mice seems to be partly due to pTreg cell deficiency since pTreg cell-deficient mice, which suffer a deletion of the CNS1 enhancer at *Foxp3*, also showed a disturbed composition of the gut microbiota [26,27]. These data indicate that Treg cells are required for establishing a normal composition of the gut microbiota. Mechanistically, the Treg-directed establishment of a healthy gut microbiota is likely mediated by IgA. Kawamoto et al. showed that Foxp3^+^ Treg cells differentiate into T follicular helper (Tfh) cells, which facilitate the production of bacteria-specific IgA in Peyer`s patches (PPs), thereby contributing to the diversification of the gut microbiota [28].

A mutualistic relationship exists between Foxp3^+^ Treg cells and commensal gut microbes: Foxp3^+^ Treg cells promote a diverse gut microbiota, and gut microbes themselves also induce Treg cells in the intestine through their metabolites. Previous studies showed that Clostridia induce colonic pTreg cells via the production of butyrate [29,30,31,32,33] and that oral administration of Clostridia strains to mice confers resistance to colitis and allergic diarrhea [29,31]. In addition, bile acid metabolites produced by gut microbes promote Treg cell differentiation through the production of mitochondrial reactive oxygen species [34].

While the role of Treg cells in the normal development of gut microbiota is well established, their role in the maintenance of the gut microbiota composition in adulthood remains poorly understood. *Foxp3*-knockout and scurfy mice develop fatal autoimmune diseases and exhibit premature death, preventing the elucidation of the roles of Treg cells in adult mice. In this study, we aimed to explore this role using *Foxp3* conditional-knockout mice (Foxp3 cKO), in which tamoxifen administration induced *Foxp3* gene deletion. Conditional knockout of *Foxp3* in adult mice resulted in a perturbed composition of the gut microbiota, and in particular, a decrease in the relative abundance of Clostridia, which are known to induce pTreg cell development in intestinal tissues. Treg cell dysfunction upon *Foxp3* deletion increased the proportion of Ig-coated fecal bacteria. This increase resulted from the leakage of Igs into the gut lumen by the loss of mucosal integrity, which was dependent on the gut microbiota. These data suggest that Treg cell dysfunction in adult mice leads to gut dysbiosis, probably through the aberrant binding of antibodies to gut microbes.

## 2. Results

### 2.1. Conditional Knockout of Foxp3 Changed the Composition of Gut Microbiota

**Figure 1:**
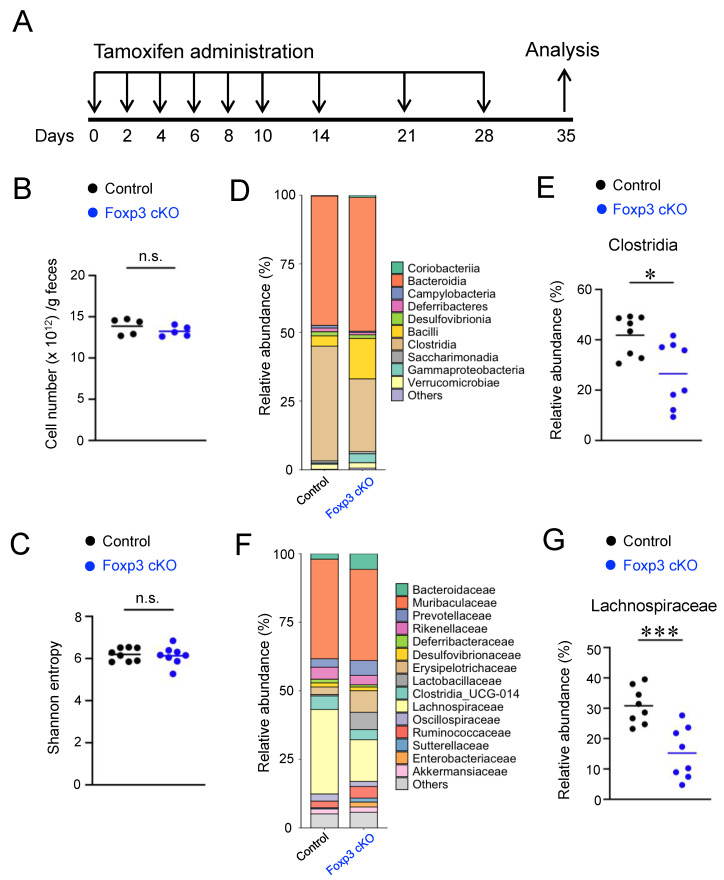
Conditional knockout (cKO) of forkhead box P3 (Foxp3) altered the composition of the gut microbiota. (**A**) Experimental design. Foxp3 cKO and control mice were orally administered tamoxifen once every 2 days until day 10 and then on days 14, 21, and 28. The mice were analyzed on day 35. (**B**) Total bacteria quantified using qPCR of the 16S rRNA gene in fecal samples. (**C**–**G**) Sequence analysis of bacterial 16S rRNA gene in fecal samples. Comparison of α-diversity (Shannon entropy) in the gut microbiota of each group (**C**). Compositions of gut microbial communities at the class level (**D**,**E**) and family level in each group (**F**,**G**). Each dot in the graphs represents an individual mouse, and horizontal lines represent the means. The data represent two (**B**) or three (**C**–**G**) independent experiments. * *p* < 0.05, *** *p* < 0.001, n.s.: not significant (Student’s *t*-test).

To study the effect of Treg dysfunction on the gut microbiota of adult mice, Foxp3 cKO and control mice were orally administered tamoxifen (Figure 1A). There was no significant difference in the number of total fecal bacteria between Foxp3 cKO and control mice (Figure 1B). To assess whether the conditional knockout of *Foxp3* affects the composition of gut microbiota, we analyzed fecal bacteria populations using 16S rRNA gene sequencing. The α-diversity measured by Shannon entropy, which indicates species richness, did not change among feces from Foxp3 cKO and control mice (Figure 1C). However, at the class level, the relative abundance of Clostridia in the phylum Firmicutes decreased in the feces of Foxp3 cKO mice compared with that in controls (Figure 1D,E). Furthermore, the relative abundance of the family Lachnospiraceae in the order Clostridiales was also considerably decreased in the feces of Foxp3 cKO mice (Figure 1F,G). Therefore, our data suggest a symbiotic relationship between Treg cells and Treg-inducing bacteria species.

### 2.2. Dysfunction of Treg Cells Increased Ig-Coated Bacteria

**Figure 2:**
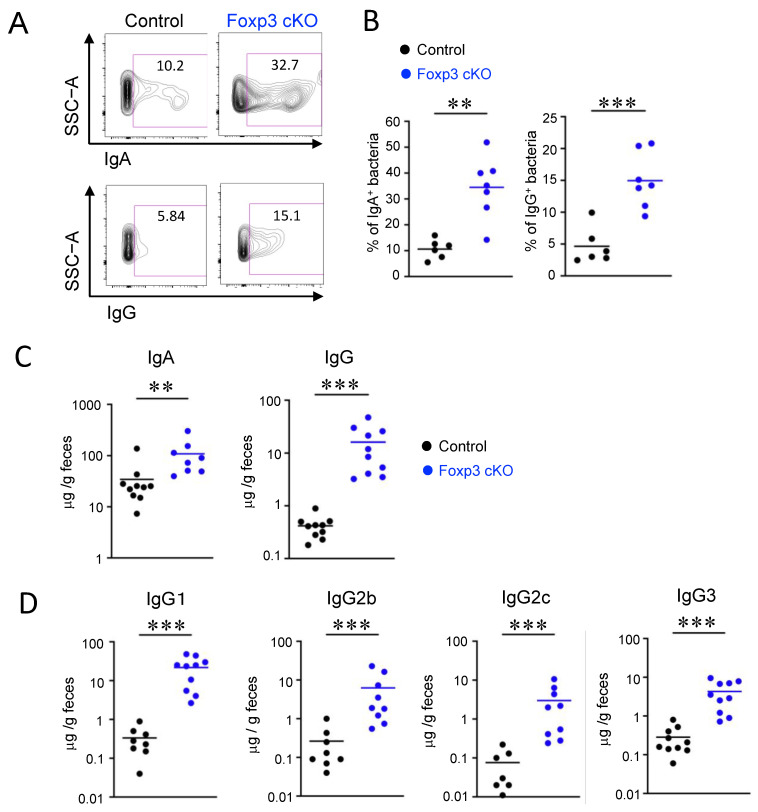
Dysfunction of regulatory T cells increased Immunoglobulin (Ig)-coated bacteria. (**A**,**B**) Flow cytometric analyses of IgA- and IgG-coated fecal bacteria. Representative flow cytometry plots (**A**) and quantification (**B**). Numbers in the plots indicate the percentages of IgA-coated (top) and IgG-coated (bottom) bacteria. (**C**) Amounts of fecal IgA and IgG (μg/g feces) measured using enzyme-linked immunosorbent assay (ELISA). (**D**) Amounts of fecal IgG1, IgG2b, IgG2c, and IgG3 (μg/g feces) measured using ELISA. Each dot in the graphs represents an individual mouse, and horizontal lines represent the means. The data represent three independent experiments. ** *p* < 0.01, *** *p* < 0.001 [Welch’s *t*-test (**B**) or Mann–Whitney test (**C**,**D**)].

To examine whether Treg dysfunction induced by *Foxp3* deletion affects the reactivity of luminal immunoglobulins to the gut microbiota, we analyzed the Ig-coated bacteria using flow cytometry. Consistent with a previous report [28], the proportion of IgA-coated bacteria in the feces of Foxp3 cKO mice was markedly increased compared to that in controls (Figure 2A,B). Unexpectedly, Foxp3 deficiency also caused a marked increase in the proportion of IgG-coated bacteria (Figure 2A,B).

To examine whether the increase in Ig-coated bacteria observed in Foxp3 cKO mice was due to an increase in Ig levels in the intestinal tract, we measured fecal immunoglobulins. Fecal IgA and IgG levels were increased in Foxp3 cKO mice compared to those in control mice (Figure 2C). The elevation of the Ig-coated bacteria and the fecal immunoglobulins was not observed until day 14 in Foxp3 cKO mice, suggesting that IgA and IgG induction in the intestinal lumen occurred later (Supplementary Figure S1A–D). Further analysis of IgG revealed that levels of all subclasses (IgG1, IgG2b, IgG2c, and IgG3) increased in the intestinal lumen of Foxp3 cKO mice (Figure 2D). These data suggested that Treg dysfunction increased immunoglobulin binding to bacteria in the gut, possibly resulting in gut dysbiosis.

### 2.3. Deletion of Foxp3 Promoted Intestinal Permeability but Did Not Increase the Production of Bacteria-Specific IgG

**Figure 3:**
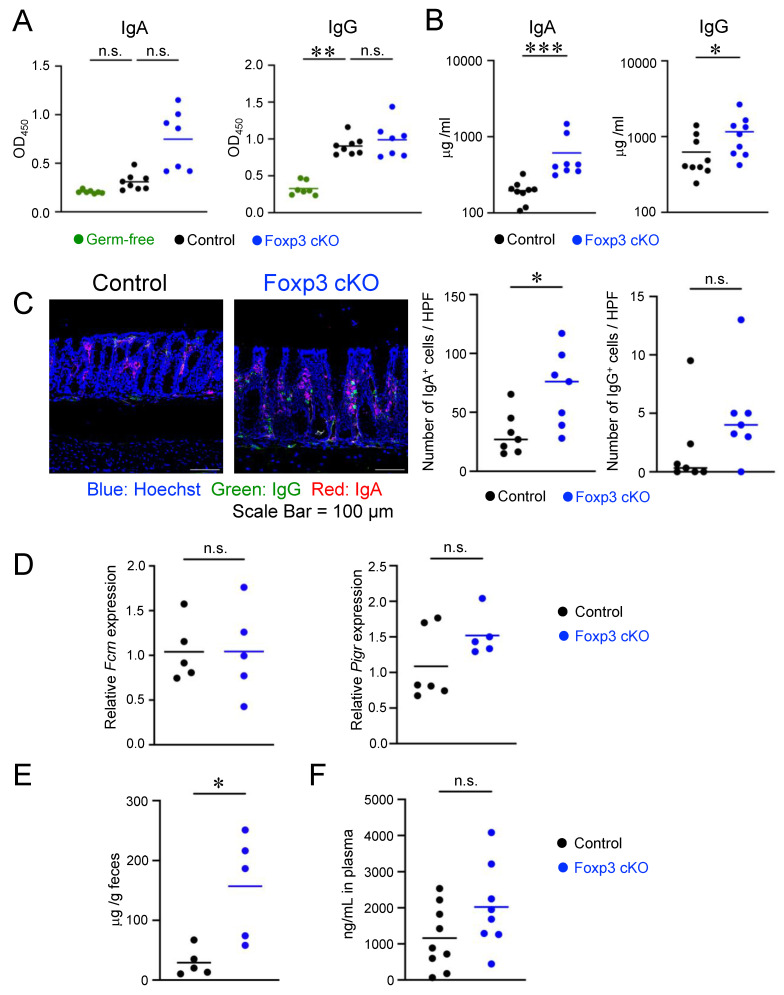
Deletion of Foxp3 promoted intestinal permeability. (**A**) Serum IgA and IgG titers specific for fecal bacteria measured using ELISA. Data represent the absorbance at 450 nm. (**B**) Serum IgA and IgG concentrations measured using ELISA. (**C**) Immunofluorescence microscopy analysis of the colon of controls (left) and Foxp3 cKO mice (right). Sections were stained for Hoechst (blue), IgG (green), and IgA (red). Scale bars indicate 100 μm. The right panels show the numbers of IgA^+^ (left) and IgG^+^ (right) cells. (**D**) Relative mRNA expressions of FcRn (Fcrn) (left) and pIgR (Pigr) (right) in the colon measured using qPCR. (**E**) Amounts of fecal albumin (μg/g feces) measured using ELISA. (**F**) Concentrations of FITC-dextran in the plasma were measured. Each dot in the graphs represents an individual mouse, and horizontal lines represent the means. The data represent two (**D**,**F**) or three (**A**–**C**,**E**) independent experiments. * *p* < 0.05, ** *p* < 0.01, *** *p* < 0.001, n.s.: not significant [Kruskal–Wallis test (**A**), Mann–Whitney test (**B**), Student’s *t*-test (**C**,**F**), or Welch’s *t*-test (**D**,**E**)].

The augmented Ig-coated bacteria in the gut of Foxp3 cKO mice prompted us to examine whether *Foxp3* deletion induced the production of bacteria-specific antibodies. To test this hypothesis, we measured the titer of bacteria-specific antibodies in the serum. As previously reported [35], anti-bacterial IgA and IgG titers were higher in controls than in germ-free mice (Figure 3A), indicating that bacterial antigens invaded the body and induced specific IgA and IgG responses in healthy mice under SPF conditions. Unexpectedly, despite the increase in IgA levels, Foxp3 cKO mice exhibited similar titers of bacteria-specific IgG as controls (Figure 3A), indicating that Treg dysfunction did not increase the production of bacteria-specific IgG. In contrast, *Foxp3* deletion extensively augmented total IgG serum titers (Figure 3B). These data suggest that the increased IgG-coated bacteria in Foxp3 cKO mice did not result from an augmented production of gut bacteria-specific antibodies.

The above-mentioned results suggest that the increased IgG-coated bacteria in Foxp3 cKO mice might result from an augmented transfer of immunoglobulins to the intestinal lumen. Since the intestinal lamina propria contains many immunoglobulin-producing cells that supply IgA and IgG to the lumen by active transport, we examined whether these cells were altered in the lamina propria of Foxp3 cKO mice using immunofluorescence microscopy. Both IgA^+^ and IgG^+^ cells showed increased tendency in the intestinal lamina propria of Foxp3 cKO mice compared to those in controls (Figure 3C).

Since FcRn expression is induced by pro-inflammatory cytokines through NF-kB activation [36,37], we tested whether *Foxp3* deletion induced FcRn expression in the intestine. No remarkable difference in the expression of *Fcrn* mRNA in intestinal tissues was found between Foxp3 cKO and control mice (Figure 3D). The pIgR expression in Foxp3 cKO and control mice were also comparable (Figure 3D).

These findings indicate that the active transfer of IgG into the gut lumen, possibly via FcRn, in Foxp3 cKO mice was not augmented although the IgG production in the gut lamina propria seemed to be enhanced. Because intestinal permeability is positively correlated with the loss of serum proteins into the intestinal lumen [38], we examined the possibility of immunoglobulin leakage from the blood into the gut lumen and measured serum albumin levels in the gut. The concentration of fecal albumin was higher in Foxp3 cKO than in control mice (Figure 3E). In addition, the serum level of orally administered FITC-dextran also showed an increasing trend in Foxp3 cKO mice compared to control mice (Figure 3F). These results suggest that leakage of immunoglobulins from tissues into the intestinal lumen occurred in Foxp3 cKO mice due to increased gut permeability.

### 2.4. Th2- and Th17-Associated Intestinal Inflammatory Responses Were Induced by Dysfunction of Treg Cells

**Figure 4:**
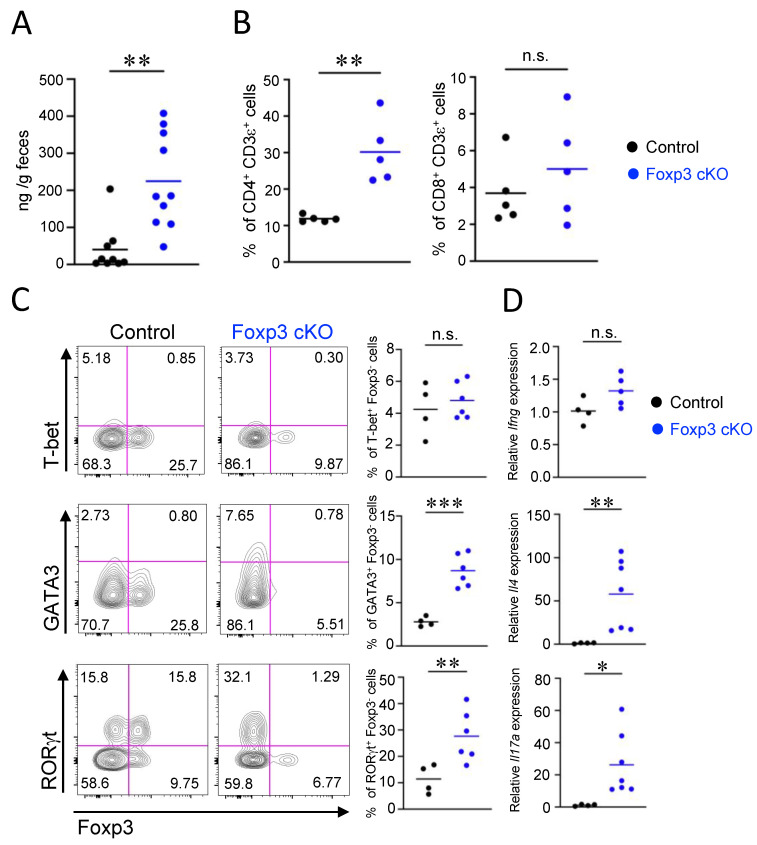
Dysfunction of regulatory T cells induced T helper (Th)2- and Th17-associated intestinal inflammatory responses. (**A**) Amounts of fecal lipocalin-2 (ng/g feces) measured using ELISA. (**B**) Frequencies of CD4^+^ CD3ε^+^ or CD8^+^ CD3ε^+^ cells in the CD45^+^ cells in the colonic lamina propria analyzed using flow cytometry. (**C**) Frequencies of T-bet^+^ Foxp3-, GATA3^+^ Foxp3-, and RORγt^+^ Foxp3- cells in the CD4^+^ CD45^+^ cells analyzed using flow cytometry. Representative flow cytometry plots (left) and quantification (right). (**D**) Relative mRNA expressions of interferon gamma (Ifng), interleukin 4 (Il4), and interleukin 17a (Il17a) in the colonic tissues measured using qPCR. Each dot in the graphs represents an individual mouse, and horizontal lines represent the means. The data represent three independent experiments. * *p* < 0.05, ** *p* < 0.01, *** *p* < 0.001, n.s.: not significant (Welch’s *t*-test).

The leakage of serum albumin observed in Foxp3 cKO mice suggests that the inflammatory response induced by Treg dysfunction caused an increase in the permeability of the gut epithelial barrier. Thus, we examined the inflammatory status of the gut and the immune cells in the lamina propria of Foxp3 cKO mice. Severe inflammation, indicated by aberrant infiltration of leukocytes and hypertrophy of muscularis mucosae, was not detected in the histological analysis of intestinal tissue sections of Foxp3 cKO mice (Supplementary Figure S2A). Although colon length was comparable between Foxp3 cKO and control mice, colon weight was markedly increased in Foxp3 cKO mice (Supplementary Figure S2B,C). In addition, the concentration of fecal lipocalin-2, a marker of intestinal inflammation, increased in Foxp3 cKO mice compared to that in control mice (Figure 4A). Flow cytometric analysis showed that the proportion of CD45^+^ leukocytes increased in the lamina propria of Foxp3 cKO mice (Supplementary Figure S2D). Foxp3^+^ Treg cells are known to suppress the proliferation of CD4^+^ helper and CD8^+^ cytotoxic T cells through direct and indirect mechanisms [39]. Thus, we examined the proportion of CD4^+^ and CD8^+^ T cells in CD45^+^ leucocytes in the lamina propria. Among CD45^+^ cells, the proportion of CD4^+^, but not CD8^+^, T cells increased in the lamina propria of Foxp3 cKO mice (Figure 4B). We next examined which types of CD4^+^ T cells were expanded in the lamina propria of Foxp3 cKO mice by intracellular staining for the master transcription factors T-bet, GATA3, RORγt, and Foxp3. It has been reported that a substantial proportion of Foxp3^+^ Treg cells express GATA3 or RORγt in the intestinal lamina propria [40,41,42,43]. Thus, we defined Th1, Th2, and Th17 cells as CD4^+^T-bet^+^Foxp3^−^, CD4^+^GATA3^+^Foxp3^−^, and CD4^+^ RORγt^+^Foxp3^−^ cells, respectively. The percentage of T-bet^+^ Foxp3^−^ Th1 cells was similar among Foxp3 cKO and control mice, whereas that of GATA3^+^ Foxp3^−^ Th2 cells increased in Foxp3 cKO mice. The percentage of RORγt^+^ Foxp3^−^ Th17 cells was markedly elevated in Foxp3 cKO mice, contrasting the striking decrease in RORγt^+^ Foxp3^+^ Treg cells (Figure 4C). We then tested the gene expression of IFNg, IL-4, and IL-17A as representative cytokines for Th1, Th2, and Th17, respectively. Consistent with the elevated proportion of Th2 and Th17 cells, the relative expression levels of *Il4* and *Il17a*, but not *Ifng*, in intestinal tissues of *Foxp3* cKO mice were increased compared to those in control mice (Figure 4D), confirming that Th2 and Th17 responses are augmented in the intestine of Foxp3 cKO mice. The intracellular staining of cytokines also revealed that IL-4- and IL-17-, but not IFNg-, producing CD4^+^ T cells were increased in Foxp3 cKO mice (Supplementary Figure S3). Collectively, our data indicate that Treg dysfunction induced by *Foxp3* deletion triggered Th2- and Th17-associated intestinal inflammatory responses, resulting in increased permeability of the gut epithelial barrier.

### 2.5. Impairment of Intestinal Barrier by Dysfunction of Treg Cells Depends on Gut Microbiota

**Figure 5:**
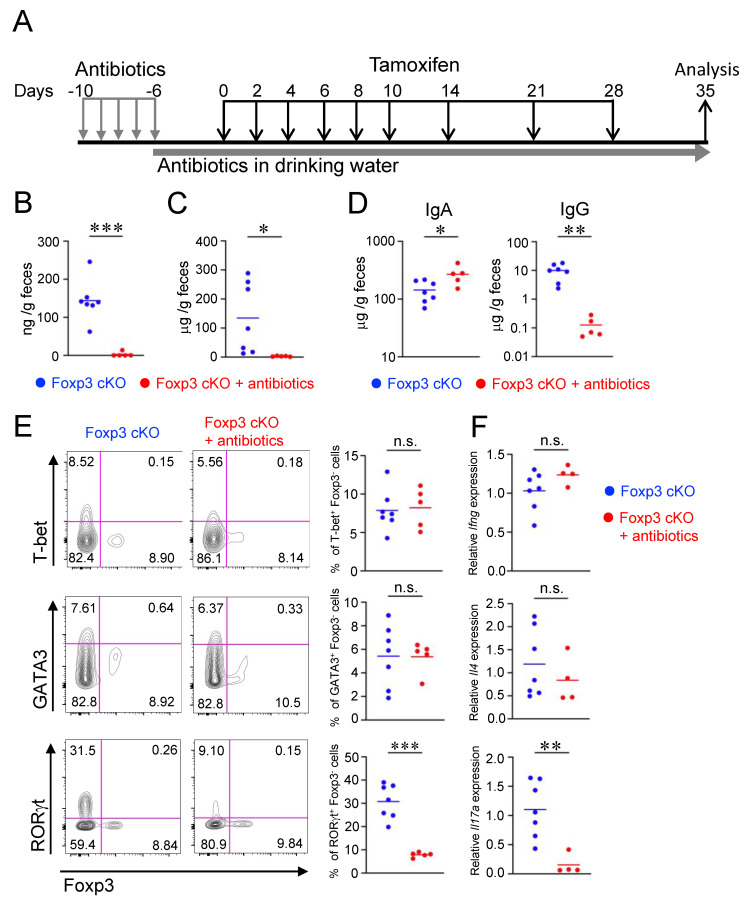
Antibiotic treatment attenuated gut barrier dysfunction and the Th17 response. (**A**) Experimental design. Foxp3 cKO mice were orally administered antibiotics once daily from days −10 to −6 of tamoxifen administration. From day −5 to experimental closure, Foxp3 cKO mice were administered antibiotics ad libitum in the drinking water. Foxp3 cKO mice were orally administered tamoxifen as shown in Figure 1A. (**B**–**D**) Amounts of fecal lipocalin-2 (**B**), fecal albumin (**C**), and fecal IgA and IgG (**D**) measured using ELISA. (**E**) Frequencies of T-bet^+^ Foxp3^−^, GATA3^+^ Foxp3^−^, and RORγt^+^ Foxp3^−^ cells in CD4^+^ CD45^+^ cells analyzed using flow cytometry. Representative flow cytometry plots (left) and quantification (right). (**F**) Relative mRNA expressions of Ifng, Il4, and Il17a in the colonic tissues measured using qPCR. Each dot in the graphs represents an individual mouse, and horizontal lines represent the means. The data represent two (**F**) or three (**B**–**E**) independent experiments. * *p* < 0.05, ** *p* < 0.01, *** *p* < 0.001, n.s.: not significant [Welch’s *t*-test (**B**,**C**,**E**,**F**) or Mann–Whitney test (**D**)].

To clarify the role of the gut microbiota in the intestinal barrier dysfunction of Foxp3 cKO mice, the mice were administered antibiotics to deplete gut microbes 10 days prior to tamoxifen administration (Figure 5A). Antibiotic treatment extensively decreased fecal lipocalin-2 and serum albumin levels in Foxp3 cKO mice (Figure 5B,C), indicating that the depletion of gut microbes suppressed the gut hyperpermeability induced by Treg dysfunction. Fecal IgG concentration was also markedly reduced by antibiotic treatment. In contrast, the fecal IgA concentration was increased in antibiotic-treated Foxp3 cKO mice compared to that in non-treated mice (Figure 5D). The serum concentrations of IgA and IgG were not affected by antibiotic treatment (Supplementary Figure S4). These data suggest that the increase in fecal IgG mediated by Treg dysfunction mainly results from the impairment of the gut epithelial barrier function. It is possible that the increase in fecal IgA in antibiotic-treated mice reflects the increase in free fecal IgA, which should bind to gut microbes in the absence of antibiotic treatment. Antibiotic treatment of Foxp3 cKO mice also reduced the percentage of RORγt^+^ Foxp3^−^ Th17 cells in the lamina propria. In contrast, the percentages of GATA3^+^ Foxp3^−^ Th2 and T-bet^+^ Foxp3^−^ Th1 cells were not reduced by antibiotic treatment (Figure 5E). Consistent with the change in the Th cell population, the relative expression of *Il17a* was markedly reduced, whereas that of *Il4* and *Ifng* remained unchanged in the intestinal tissues of antibiotic-treated Foxp3 cKO mice compared to those in non-treated mice (Figure 5F). These data suggest that gut microbiota-dependent activation of the Th17 response may be responsible for the gut barrier dysfunction in Foxp3 cKO mice.

## 3. Discussion

In this study, we demonstrated that Treg cell dysfunction leads to intestinal dysbiosis, with a particularly remarkable reduction in the proportion of Lachnospiraceae belonging to Clostridium cluster XIVa, known to induce Treg cells. Furthermore, we observed increased levels of fecal immunoglobulins and Ig-coated bacteria in Foxp3 cKO mice, resulting from an impaired gut epithelial barrier function, which is dependent on intestinal bacteria.

The mammalian intestinal tract is colonized by bacteria that have coevolved with the host in a symbiotic relationship. These commensal bacteria play beneficial roles in the host, such as the supply of nutrient metabolites and the protection against pathogenic organisms. Thus, the host immune system must maintain tolerance against commensal bacteria. A breakdown of this tolerance leads to dysregulated immune responses against gut microbiota, resulting in intestinal inflammation. Foxp3^+^ Treg cells are considered to play a central role in establishing and maintaining tolerance to intestinal bacteria [44]. Additionally, the intestinal Treg cell population is also influenced by the gut microbiota [22]. Moreover, germ-free mice exhibit a marked reduction in the Treg cell population in their colonic lamina propria, and microbiota colonization induces colonic Treg cells in these mice [29,45]. Among intestinal microbes, Clostridia induce pTreg in the intestinal tissue by producing butyrate [29,30,31,32]. On the other hand, scurfy mice exhibit gut dysbiosis characterized by decreased Firmicutes and increased Bacteroidetes proportions [24]. A similar dysbiosis of the gut microbiota is observed in pTreg-deleted mice [26]. Kawamoto et al. reported that the transfer of Foxp3^+^ Treg cells into T cell-deficient mice promoted the diversification of Firmicutes, including Clostridium clusters IV and XIVa [28]. Although previous studies using Treg-deficient mouse models showed that Treg cells are necessary for the normal development of gut microbiota [24,26], the role of Treg cells in the maintenance of gut microbiota composition in adulthood has not been investigated because these mouse models are deficient in Treg cells since birth. In this study, we demonstrated that the deletion of *Foxp3* in adult mice induced a change in the composition of the intestinal microbiota, indicating that Treg cells are required for not only the normal development of microbiota but also the maintenance of their normal composition in adulthood. In addition, we showed a marked reduction in the proportion of Lachnospiraceae in Firmicutes bacteria in Foxp3 cKO mice. It has been shown that Treg deficiency caused a reduction in Firmicutes at the phylum level [24,26]. However, among the phylum Firmicutes, the specific bacteria affected at the family level in Treg-deficient mice have not been demonstrated. Given that Lachnospiraceae produces Treg-inducing butylate [30,31], our data suggest a strong relationship between Treg and Lachnospiraceae in the gut immune homeostasis. Taken together, these data suggest that not only can specific commensal bacteria induce pTreg cells but Treg cells themselves are also required for the expansion and maintenance of these bacteria. Thus, it is possible that a decrease in intestinal Treg cells reduces pTreg-inducing bacteria, establishing a vicious cycle that leads to a further decrease in Treg cells in intestinal tissues.

Humoral immune response, particularly secretory IgA, plays an important role in maintaining the homeostasis of the intestinal microbiota. T cell-dependent IgA class switching occurs primarily in PPs where Tfh cells assist antigen-specific B cells. Previous studies have revealed that Treg cells differentiate into Tfh cells in PPs where they efficiently induce IgA production [46]. Furthermore, T follicular regulatory (Tfr) cells also facilitate the diversification of gut microbiota through the diversification and selection of IgA [28]. Therefore, Foxp3^+^ Treg and/or Tfr cells contribute to the establishment and maintenance of the diversified gut microbiota via IgA production. In the absence of Foxp3^+^ T cells, the proportion of IgA-coated bacteria increases with the decrease in the affinity maturation of IgA, which leads to a reduction in the diversity of intestinal microbes, especially those of the phylum Firmicutes [28]. Consistent with these observations, Foxp3 cKO mice exhibited an increased proportion of IgA-coated bacteria, suggesting that the aberrant binding of IgA to the intestinal microbes leads to dysbiosis in these mice.

In addition to IgA-coated bacteria, Foxp3 cKO mice showed an increased proportion of IgG-coated bacteria. IgG is the most abundant immunoglobulin isotype in blood, and a small amount is present in the intestinal lumen. IgG produced from IgG-positive plasma cells is transported to the intestinal lumen via FcRn expressed on the intestinal epithelium [47]. Although we observed an increase in IgG-positive cells in the intestinal lamina propria of Foxp3 cKO mice, FcRn expression was not augmented in the intestinal tract. Instead, serum albumin and lipocalin-2 levels in the intestinal lumen were increased. In addition, the increase of serum albumin and IgG in the intestinal lumen was suppressed by antibiotic treatment. These data indicate that the intestinal inflammation triggered by gut microbes leads to a dysfunctional epithelial barrier that allows non-selective leakage of blood IgG into the intestinal lumen, rather than enhanced selective transport. In the intestinal lumen, IgG is considered to protect the host from pathogenic bacteria by promoting their elimination mediated by neutrophils in the intestinal lumen [15]. However, it remains unknown whether IgG binding to commensal bacteria affects gut microbial composition. Studies have reported an increased proportion of IgG-coated bacteria in the feces of active IBD patients who present intestinal barrier disruption, which correlates with disease severity [48,49,50]. A reduction in the diversity of gut microbiota and the proportion of Firmicutes in patients with IBD has also been reported [51,52]. Therefore, it is possible that aberrant IgG binding to gut microbes leads to dysbiosis, further exacerbating the disease. Further investigations are warranted to clarify the role of IgG in the development of dysbiosis of gut bacterial communities during inflammation.

The development of spontaneous colitis in IL-10-deficient mice is not observed under germ-free conditions [53]. In addition, antibiotic treatment prevents colitis in these mice, as well as in other mouse models of IBD [54,55,56], indicating that the gut microbiota is involved in the development of IBD. In a previous report, the intestinal inflammation in Treg-depleted mice ameliorated in the absence of gut microbiota [57]. We found that the removal of intestinal bacteria by antibiotic treatment suppressed intestinal inflammation and increased fecal IgG levels in Foxp3 cKO mice. Antibiotic treatment has been reported to suppress systemic inflammation and prolong the lifespan of scurfy mice [24]. Therefore, it is possible that the combination of Treg cell dysfunction and stimuli derived from intestinal bacteria may have induced intestinal inflammation in Foxp3 cKO mice, resulting in IgG leakage from the blood.

We demonstrated that Th2 and Th17 responses were augmented in the intestine of Foxp3 cKO mice and that antibiotic treatment suppressed Th17, but not Th2, response. In addition, antibiotic treatment suppressed the intestinal inflammation and permeability of Foxp3 cKO mice. Considering these data, Th17 cells are possibly responsible for the impairment of the gut epithelial barrier function induced by Treg cell dysfunction. An enrichment in Th17 cells and an upregulation of Th17-associated cytokines have been observed in the intestinal tissues of patients with IBD [58,59,60]. Accordingly, IL-17A and IL-17R knockout mice are resistant to dextran sulfate sodium (DSS)- and 2,4,6-trinitrobenzene sulfonic acid-induced colitis, respectively [61,62]. However, the role of IL-17 in colitis is controversial. The blockade of IL-17A by anti-IL-17A antibodies aggravates disease severity in DSS-induced colitis [63,64]. Adoptive transfer of T cells deficient in Th17-associated cytokines (IL-17A, IL-17F, and IL-22) into Rag1 knockout mice induced severe colitis comparable to that induced by wild-type cells [65]. Interestingly, the adoptive transfer of T cells deficient in RORγt failed to induce colitis, whereas recombinant IL-17A administration induced severe colitis in mice transferred with RORγt knockout T cells [65], suggesting a crucial role of Th17 cells in the pathogenesis of colitis. Further studies are necessary to determine whether Th17 cells are essential to the impairment of the gut epithelial barrier function induced by Treg cell dysfunction.

In summary, this study demonstrated that Treg cell dysfunction in adult mice impairs the gut epithelial barrier function, resulting in an aberrant elevation of anti-microbial antibodies in the intestinal lumen, dependent on the gut microbiota. Our data also indicate that Treg cells are required for the maintenance of the normal composition of the gut microbiota, especially Treg-inducing bacteria species. Although the underlying mechanism remains unclear, anti-microbial antibodies that leak into the intestinal lumen possibly contribute to intestinal dysbiosis.

## 4. Materials and Methods

### 4.1. Mice

The generation of *Foxp3*^flox^ *Rosa26*^CreERT2^ C57BL/6 mice was performed as previously described [66]. Mice aged 3 to 4 months were fed FR-1 chow (Funabashi Farm, Funaba, Japan) and maintained under specific pathogen-free (SPF) conditions at the animal facilities of the Tokyo University of Science (Chiba, Japan). At the end of the experiments, the mice were sacrificed via carbon dioxide (CO_2_) inhalation.

### 4.2. Tamoxifen Administration

Foxp3 cKO and control mice were orally administered 2 mg tamoxifen (Cayman Chemical, Ann Arbor, MI, USA) solubilized in corn oil (Wako, Osaka, Japan) once per day, on days 0, 2, 4, 6, 8, 10, 14, 21, and 28.

### 4.3. Antibiotic Treatments

Foxp3 cKO mice were administered 300 μL of antibiotic cocktail (8.0 g/L neomycin, 8.0 g/L ampicillin, 4.0 g/L vancomycin, and 8.0 g/L metronidazole) via oral gavage once daily, from day −10 to day −6 of tamoxifen administration. From day −5 to experimental closure, Foxp3 cKO mice were treated with an antibiotic cocktail (1.0 g/L neomycin, 1.0 g/L ampicillin, 0.5 g/L vancomycin, and 1.0 g/L metronidazole), with *ad libitum* access to drinking water containing antibiotics.

### 4.4. Isolation and Stimulation of Cells from Lymphoid Tissues

To prepare colonic lamina propria cells, colons were harvested from sacrificed mice and dissociated from fat. Colonic tissues were opened longitudinally, and the intestinal contents were gently removed. Tissues were cut into 2 cm pieces and washed in ice-cold PBS. To remove the epithelium, tissues were incubated in extracting solution (Hank’s Balanced Salt Solution containing 10 mM HEPES, 20 mM EDTA, and 0.1 mM 2-mercaptoethanol) at 37 ℃ for 20 min. After vortexing and washing with 2% FCS in RPMI 1640 medium, tissues were finely minced and incubated in an enzyme solution [RPMI 1640 medium containing 2% FCS, 1 mg/mL collagenase (Wako), and 0.25 mg/mL DNase I (Sigma-Aldrich, St. Louis, MO, USA)] at 37 °C for 20 min. Afterward, the digested tissues were passed through a 100 μm cell strainer and centrifuged at 400× *g* for 5 min. The precipitates were resuspended in 40% Percoll (Cytiva, Uppsala, Sweden) and centrifuged at 1200× *g* for 15 min. Sediments were washed with 2% FCS in RPMI 1640 medium and used for analysis of colonic lamina propria cells. For intracellular cytokine staining, colonic lamina propria cells were stimulated with 50 ng/mL PMA (Sigma-Aldrich) and 500 ng/mL ionomycin (Cayman chemical) in the presence of 2 μM monensin (Biolegend, San Diego, CA, USA) at 37 °C for 4 h.

### 4.5. Flow Cytometry

For surface and intracellular staining, non-specific binding was blocked with an anti-CD16/32 antibody (CD16/32; Biolegend) for 10 min prior to staining with the following antibodies: anti-CD4 (RM4-5), anti-CD3e (17A2), anti-CD45 (30F-11), anti-T-bet (4B10), anti-B220 (RA3-6B2), anti-CD8a (53-6.7), and anti-IL-17 (TC11-18H10.1) from BioLegend; anti-GATA3 (L50-823), anti-RORgt (Q31-378), anti-IFNg (XMG1.2), and anti-IL-4 (11B11) from BD Biosciences; anti-IgG and anti-Foxp3 (FJK-16s) from Invitrogen; and anti-IgA from Southern Biotech. For intracellular staining, lymphocytes were fixed, permeabilized, and stained with monoclonal antibodies using the Foxp3/Transcription Factor Staining Buffer Set (Thermo Fisher Scientific, Waltham, MA, USA) or Cytofix/Cytoperm and Perm/Wash (BD Biosciences, San Jose, CA, USA), according to the manufacturer’s instructions. To exclude dead cells, 7-AAD (Tonbo Biosciences, San Diego, CA, USA) or Fixable Viability Staining 506 (Thermo Fisher Scientific) was used. For the detection of Ig-coated bacteria, fecal samples were homogenized in PBS (1 mL/100 mg feces) containing 0.05% cOmplete (Roche, Basel, Switzerland) and centrifuged at 12,000× *g* for 5 min. The pellet was resuspended in 1% bovine serum albumin (BSA) in PBS and centrifuged at 100× *g* for 5 min. The filtered supernatant was resuspended in 1% BSA in PBS and centrifuged at 12,000× *g* for 5 min. The pellet containing fecal bacteria was stained with SYTO™ BC Green (Thermo Fisher Scientific), followed by staining with anti-Ig antibodies. Samples were analyzed using Canto II, Lyric, and Aria III flow cytometers (BD Biosciences) and the FlowJo software version 10 (BD Biosciences).

### 4.6. Enzyme-Linked Immunosorbent Assay

To measure Ig levels, 96-well ELISA plates were coated with goat anti-mouse IgA, IgG, IgG1, IgG2b, IgG2c, or IgG3 (BETHYL Laboratories, Waltham, MA, USA) overnight at 4 °C and blocked with 1% BSA in PBS. The diluted serum in 1% BSA in PBS was added to the wells and incubated for 2 h at 37 °C. To measure the specific Ig levels of fecal bacteria, fecal samples were homogenized in PBS (1 mL/100 mg feces) containing 0.05% cOmplete (Roche) and centrifuged at 12,000× *g* for 5 min; the supernatant was collected as the fecal extract. Subsequently, 96-well ELISA plates were coated with the fecal extracts in 1% BSA in PBS overnight at 4 °C and then blocked with 1% BSA in PBS. The diluted serum in 1% BSA in PBS was added to each well and incubated for 2 h at 37 °C. After washing, horseradish peroxidase (HRP)-conjugated goat anti-mouse IgA, IgG, IgG1, IgG2b, IgG2c, or IgG3 (BETHYL Laboratories) in 1% BSA in PBS was added to the wells and incubated for 1 h at 37 °C. After washing, HRP enzymatic activity was visualized by adding chromogenic TMB substrate (Biolegend) to quantify antibody titers. The reaction was stopped via the addition of 1 M H_2_SO_4_. The absorbance was measured at 450 nm using an Infinite F50R (Tecan, Männedorf, Switzerland). To detect albumin in fecal extracts, 96-well ELISA plates were coated with goat anti-mouse albumin (BETHYL Laboratories) and blocked with 1% BSA in PBS. Further, diluted fecal extracts in 1% BSA in PBS were added and incubated for 2 h at 37 °C. After washing, plates were incubated with an anti-mouse albumin antibody biotinylated using an EZ-Link™ Sulfo-NHS-LC-Biotinylation kit (Thermo Fisher Scientific) according to the manufacturer’s instructions. After reaction with HRP conjugated with streptavidin for 30 min at 37 °C, HRP enzymatic activity was visualized as described earlier. Lipocalin-2 levels in fecal extracts were measured using a Mouse Lipocalin-2/NGAL kit (R&D SYSTEMS, Minneapolis, MN, USA) according to the manufacturer’s instructions.

### 4.7. Histological Analysis

Intestinal tissues were fixed in 4% paraformaldehyde (Nacalai tesque, Kyoto, Japan) in PBS overnight at 4 °C and embedded in paraffin (Sakura FinTek, Torrance, CA, USA). After deparaffinization, tissue sections were stained with hematoxylin and eosin (Muto Pure Chemicals, Tokyo, Japan).

### 4.8. Fluorescent Immunohistochemistry

Intestinal tissues were fixed in 4% paraformaldehyde in PBS overnight at 4 °C. Fixed sections were incubated with 0.3% (*v*/*v*) Triton X-100 (Nacalai tesque) in PBS. After blocking with 3% BSA, sections were incubated with Alexa Fluor 488-conjugated anti-IgG (Southern Biotech, Birmingham, AL, USA), Alexa Fluor 647-conjugated anti-IgA (Southern Biotech), and Hoechst 33342 (Thermo Fisher Scientific) overnight at room temperature. Immunofluorescence data were obtained and analyzed using a confocal laser scanning microscope (TCS SP8; Leica, Wetzlar, Germany).

### 4.9. Quantitative Reverse Transcription Polymerase Chain Reaction (RT-qPCR)

Total RNA from tissues was extracted using TRI Reagent (Sigma-Aldrich), according to the manufacturer’s instructions. Reverse transcription into cDNA was performed using the ReverTra Ace qPCR RT Master Mix with gDNA Remover (TOYOBO, Osaka, Japan) according to the manufacturer’s instructions. qPCR was performed using a CFX Connect Real-Time PCR Detection System (Bio-Rad Laboratories, Hercules, CA, USA) and a TUNDERBIRD SYBR qPCR Mix (TOYOBO) according to the manufacturer’s instructions. The primers used were as follows: *Actb* 5′-GATCTGGCACCACACCTTCT-3′ (forward), 5′-GGGGTGTTGAAGGTCTCAAA-3′ (Reverse); *Il4*: 5′-CATCGGCATTTTGAACGAG-3′ (forward), 5′-CGAGCTCACTCTCTGTGGTG-3′ (Reverse); *Ifng*: 5′-GCGTCATTGAATCACACCTG-3′ (forward), 5′-ACCTGTGGGTTGTTGACCTC-3′ (Reverse); *Il17a* 5′-ACTACCTCAACCGTTCCACG-3′ (forward), 5′-CTTCCCAGATCACAGAGGGA-3′ (Reverse); *Fcrn* 5′-CAGCCTCTCACTGTGGACCTAGA-3′ (forward), 5′-TCGCCGCTGAGAGAAAGC-3′ (Reverse); and *Pigr* 5′-GCTCCAAAGTGCTGTTCTCC-3′ (forward), 5′-TTGCTGTGTGTCTGGAGAGG-3′ (Reverse). Relative RNA expression levels were calculated using the ΔΔCt method and normalized to Actb mRNA levels.

### 4.10. 16S rRNA Gene Sequence

Fecal samples were collected from *Foxp3*^flox^ (control) and *Foxp3*^flox^ *Rosa26*^CreERT2^ (Foxp3 cKO) mice that were co-housed from birth. Bacterial DNA was extracted from fecal samples using magLEAD (Precision System Science, Matsudo, Japan) with the E.Z.N.A. Stool DNA Kit (OMEGA Bio-tek, Norcross, GA, USA) according to the manufacturer’s instructions. The hypervariable V3–V4 region of the *16S* gene was amplified using Ex Taq Hot Start (Takara Bio, Kusatsu, Japan) and subsequently purified using AMPure XP (Beckman Coulter, Brea, CA, USA). Mixed samples were prepared by pooling approximately equal amounts of each amplified DNA and sequenced using the Miseq Reagent Kit V3 (600 Cycle) and Miseq sequencer (Illumina, San Diego, CA, USA) according to the manufacturer’s instructions. The sequence data were analyzed using Qiime2 (version 2021.4) [67]. The primer region was excluded from the raw sequences using Cutadapt in the Qiime2 plugin [68]. To obtain the sequences of representative amplicon sequence variants, the primer-free sequences were processed using DADA2 [69]. Taxonomic assignments were performed using BLAST [70] with the SILVA database (version 138) [71] for each amplicon sequence variant-representative sequence. The sequences of typical amplicon sequence variants and the abundance of these variants were extracted using the feature table [72].

### 4.11. Quantification of Fecal Bacteria

Quantification of extracted bacterial DNA from fecal samples was performed using the CFX Connect Real-Time PCR Detection System (Bio-Rad Laboratories) and SYBR Premix EX Taq II (Takara Bio) according to the manufacturer’s instructions. The following primers were used: *16S rRNA* forward 5′-CATCGGCATTTTGAACGAG-3′ and reverse 5′-CGAGCTCACTCTCT GTGGTG-3′.

### 4.12. In Vivo Intestinal Permeability Assay

Intestinal permeability was determined using fluorescein isothiocyanate (FITC)-dextran assay. FITC-dextran (4 kDa; TdB Labs, Uppsala, Sweden) was dissolved in PBS (80 mg/mL) and orally administered to mice (600 mg/kg body weight). Blood was collected 4 h after FITC-dextran administration. Plasma was extracted from the blood through centrifugation at 4 °C for 10 min at 1000× *g*. Fluorescence intensity was analyzed using a microplate reader (ARVO MX; PerkinElmer, Waltham, MA, USA) at an excitation wavelength of 485 nm and emission wavelength of 535 nm. The FITC-dextran concentration of each mouse was detected based on the FITC-dextran standard curve.

### 4.13. Statistical Analysis

For statistical analyses of two groups, the Mann–Whitney test, unpaired two-tailed Student’s *t*-test, or Welch’s *t*-test was used. For more than two groups, data were analyzed using the Kruskal–Wallis test. Differences in *p*-values < 0.05 were considered statistically significant. Statistical analyses were performed using GraphPad Prism 9 software (GraphPad Software, Inc., San Diego, CA, USA).

## Data Availability

Bacterial sequencing data in this study was deposited in an NCBI open access Sequence Read Archive database with accession number PRJNA853856.

## Supplementary Materials

The supporting information can be downloaded at: https://www.mdpi.com/article/10.3390/ijms24108549/s1.
