# Supplementary material for: Dysfunction of Foxp3+ Regulatory T Cells Induces Dysbiosis of Gut Microbiota via Aberrant Binding of Immunoglobulins to Microbes in the Intestinal Lumen
Source: Int J Mol Sci. 2023 May 10;24(10):8549. doi: 10.3390/ijms24108549 (PMC10218244; doi:10.3390/ijms24108549)

Supplementary Figure S1. Ig-coated bacteria and fecal Igs did not increase until day 14 in Foxp3 cKO mice.

(A, B) Flow cytometric analyses of IgA- and IgG-coated fecal bacteria on days 0 (A) and 14 (B). Numbers in the plots indicate the percentages of IgA-coated (left) and IgG-coated (right) bacteria. (C, D) Amounts of fecal IgA (left) and IgG (right) on days 0 (C) and 14 (D) measured using ELISA. Each dot in the graphs indicates an individual mouse, and horizontal lines represent the means. The data represent two independent experiments. n.s.: not significant [Welch's t-test (A, B) or Mann-Whitney test (C, D)].

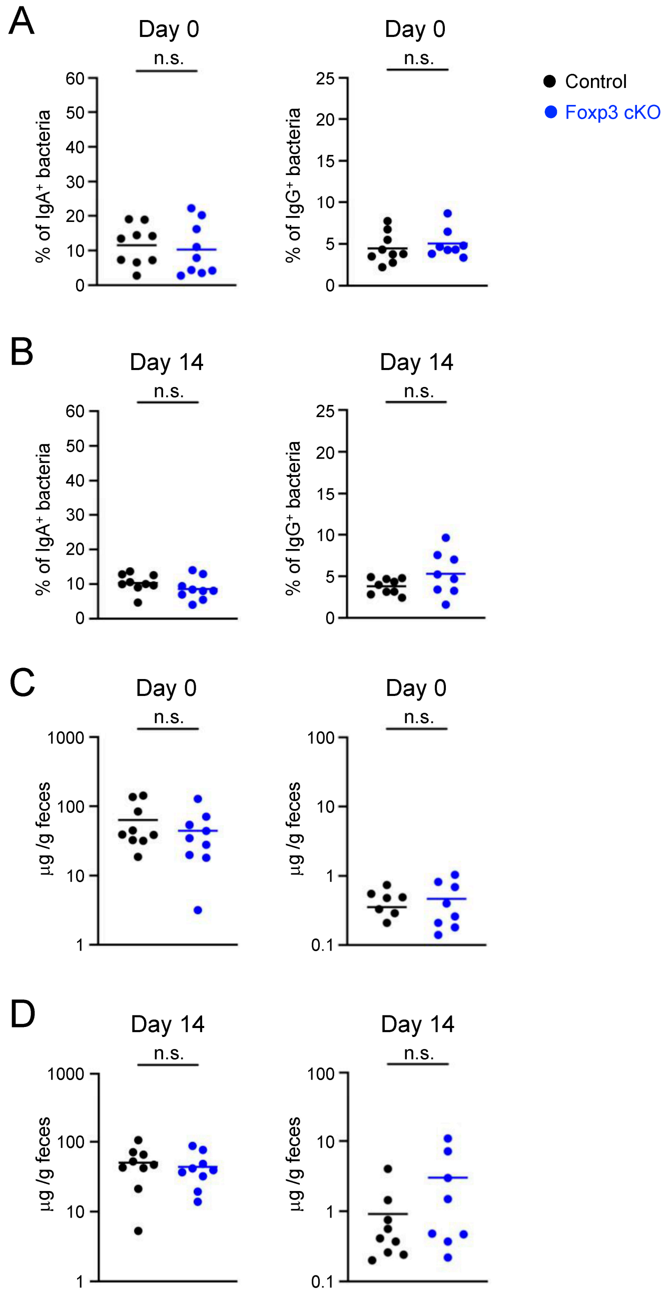

Supplementary Figure S2. Inflammatory status of intestines of Foxp3 cKO mice.

(A) Histological sections of hematoxylin and eosin (H&E)-stained colons. The scale bar indicates 100 μm. (B) Colon length and (C) weight. (D) Proportion of CD45$^+$ cells among live cells. The data represent two (A–C) or three (D) independent experiments. $^{**}p < 0.01$, $^{***}p < 0.001$, n.s.: not significant [Student's t-test (B, C) or Welch's *t*-test (D)].

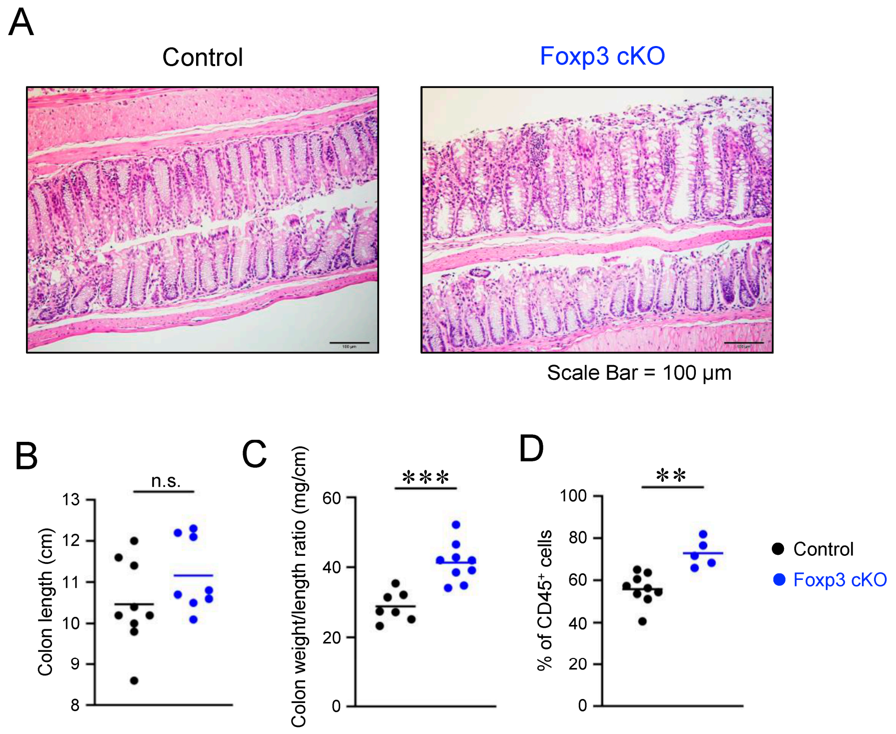

Supplementary Figure S3. Cytokine production of CD4+ T cells in lamina propria.

Lamina propria cells isolated from the colon were stimulated with PMA and ionomycin in the presence of monensin for 4 h at 37 °C. Frequencies of IFNg+, IL-4+, and IL-17+ cells among CD4+ cells were analyzed using flow cytometry. Representative flow cytometry plots (left) and quantification (right) are presented. The data represent two independent experiments. *p < 0.05, ***p < 0.001, n.s.: not significant (Welch's *t*-test).

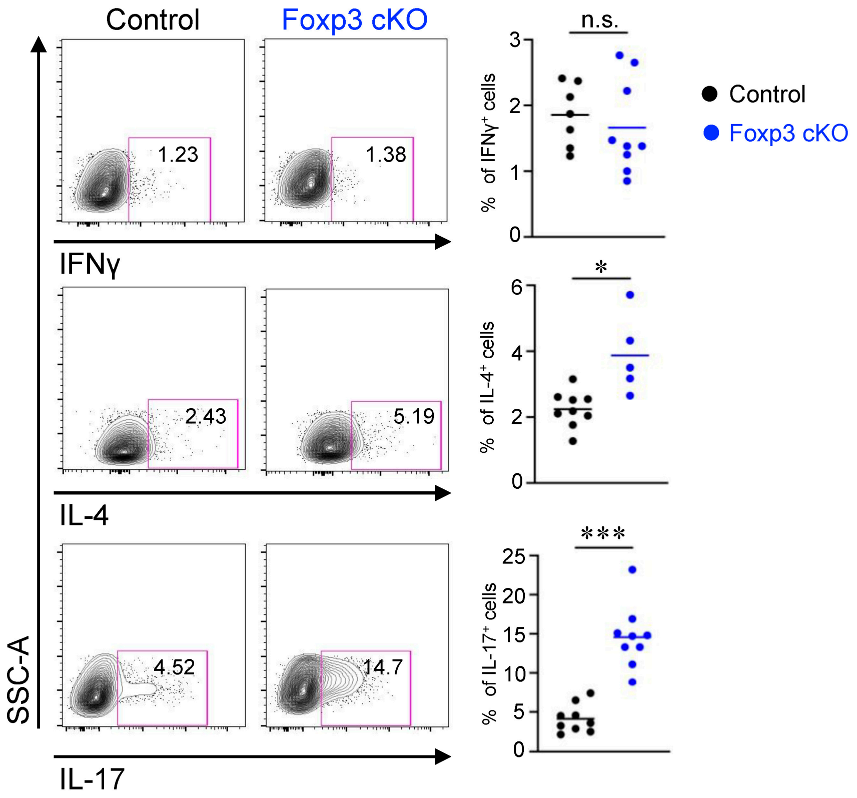

Supplementary Figure S4. Antibiotic treatment did not affect serum immunoglobulin concentrations.

Serum IgA and IgG concentrations measured using ELISA. Each dot in the graphs represents an individual mouse, and horizontal lines represent the means. The data represent three independent experiments. n.s.: not significant (Mann-Whitney test).

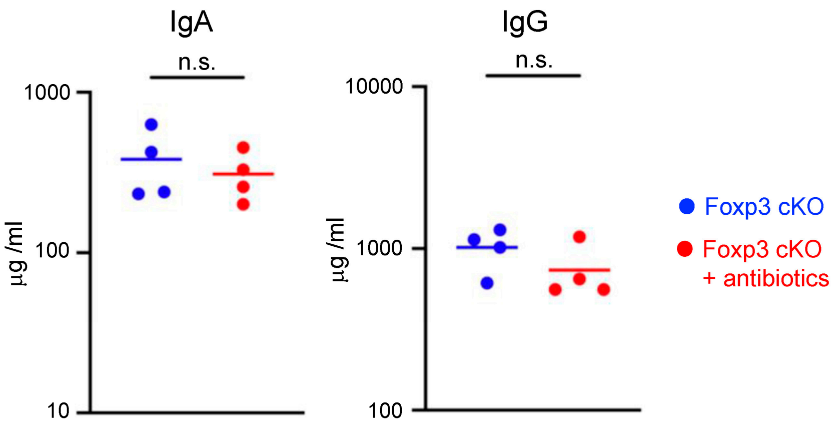

Supplement: Supplementary file 1 [file ijms-24-08549-s001.zip › ijms-2367049-Supplementary Materials.pdf]
